# Precompetitional Weight Reduction Modifies Prooxidative-Antioxidative Status in Judokas

**DOI:** 10.1155/2019/2164698

**Published:** 2019-07-22

**Authors:** Katarzyna Knapik, Karolina Sieroń, Ewa Wojtyna, Grzegorz Onik, Ewa Romuk, Ewa Birkner, Agata Stanek, Aleksandra Kawczyk-Krupka, Ryszard Plinta, Aleksander Sieroń

**Affiliations:** ^1^School of Health Sciences in Katowice, Department of Physical Medicine, Chair of Physiotherapy, Medical University of Silesia in Katowice, Poland; ^2^Institute of Psychology, University of Silesia, Katowice, Poland; ^3^Department of Biochemistry, School of Medicine with the Division of Dentistry in Zabrze, Medical University of Silesia in Katowice, Poland; ^4^School of Medicine with the Division of Dentistry in Zabrze, Department of Internal Medicine, Angiology, and Physical Medicine, Medical University of Silesia in Katowice, Poland; ^5^School of Health Sciences in Katowice, Department of Adapted Physical Activity and Sport, Chair of Physiotherapy, Medical University of Silesia in Katowice, Poland; ^6^Department of Physiotherapy, Jan Dlugosz University in Czestochowa, Poland

## Abstract

**Objective:**

The main aim of the study was an assessment of the influence of rapid weight loss on oxidative stress parameters in judokas differing in weight reduction value.

**Materials and Methods:**

The study included 30 judokas with an age range of 18-30 years (mean age: 22.4 ± 3.40 years). Enzymatic and nonenzymatic antioxidative markers, lipid peroxidation markers, and total oxidative stress were assessed three times: one week before a competition (the first stage), after gaining the desired weight (the second stage), and one week after the competition (the third stage).

**Results:**

Between the first and the second stage, the concentration of lipid hydroperoxides (LPH) decreased significantly. The superoxide dismutase (SOD), copper- and zinc-containing superoxide dismutase (Cu,Zn-SOD), ceruloplasmin (CER), malondialdehyde (MDA), LPH, and total oxidative stress (TOS) concentrations were the lowest one week after the competition. Linear regression indicated that the emphases on increased weight reduction increased the activity of glutathione peroxidase (GPx), glutathione reductase (GR), glutathione S-transferase (GST), and protein sulfhydryl (PSH) between the first and the second stage of the study. Moderate weight reduction (2-5%) resulted in elevated levels of SOD, Mn-SOD, LPH, MDA, and TOS in comparison to low and high reductions. An opposite relation was observed in PSH. In judokas, the precompetitional weight reduction range was 0.44-6.10% (mean: 2.93% ± 1.76%) of the initial body weight. Concentrations of superoxide dismutase (SOD; *p* < .01), manganese-dependent superoxide dismutase (Mn-SOD; *p* < .001), and ceruloplasmin (CER; *p* < .05) decreased between the first and the third stage of the study as well between the second and third one. Before competitions, a decrease in lipid hydroperoxide (LPH; *p* < .01) concentration was observed. A reduction of malondialdehyde (MDA; *p* < .05), LPH (*p* < .01), and total oxidative stress (TOS; *p* < .05) levels between the first and the final stage occurred. The increase in weight reduction was linearly correlated with the rise of glutathione peroxidase (GPx; *p* < .05), glutathione reductase (GR; *p* < .05), glutathione S-transferase (GST; *p* < .05), and protein sulfhydryl (PSH; *p* < .05) concentrations between the first and the second stage of the study. Moderate weight reduction (2-5%) resulted in elevated levels of SOD (*p* < .05), Mn-SOD (*p* < .05), LPH (*p* < .05), MDA (*p* < .05), and TOS (*p* < .05) in comparison to low and high reductions. An opposite relation was observed in PSH (*p* < .005).

**Conclusions:**

The effect of weight reduction in judo athletes on prooxidative-antioxidative system diversity depends on the weight reduction value.

## 1. Introduction

In combat sports, including judo, division into weight classes is obligatory, and that is why athletes reduce their weight rapidly before competitions. Rapid weight loss is aimed at qualifying to a lower category. Athletes have to ensure that they can compete with lighter athletes [1–4]. As a consequence, most athletes reduce about 2-10% of their body weight within a couple of days [5]. Athletes apply various methods to gain the desired weight, such as increasing training frequency and intensity, fasting, consuming a low-calorie diet, reducing liquid ingestion, and using plastic suits and sauna [1–4]. However, the weight loss is temporary. After qualifying, athletes abandon further weight reduction and thus regain their precompetition weight [6].

Rapid weight loss results in various physiological alterations. As dehydration is the main mechanism of weight reduction, it leads to decreased plasma volume [7, 8], glycogen depletion [9], hydroelectrolytic disturbances, impaired thermoregulation [10], and diminished concentrations of sex hormones, insulin, thyroxine, and triiodothyronine as well elevated levels of cortisol and corticotrophin [11]. What is more, habits associated with rapid weight loss, in particular, dietary restriction [12], body overheating [13], and excessive physical effort [14], may lead to prooxidative-antioxidative balance disturbances.

To our knowledge, only four studies assessed the influence of rapid weight loss on oxidative stress parameters in combat sports. However, these reports provide nonexhaustive results. Yanagawa et al. [15] reported an increased concentration of urinary markers of nucleic acid oxidation in wrestlers in response to rapid weight loss, whereas Finaud et al. [16] observed that in judokas dietary restrictions do not influence lipid peroxidation but increase urinary acid concentration. Reljic et al. [17] indicated that precompetition weight loss does not modify GSH and GSSG concentrations in boxers, while Kowatari et al. [18] proved that significant precompetition dietary restrictions do not augment neutrophil-mediated reactive oxygen species production but decrease neutrophil phagocytic activity in judokas.

Our study was conducted during a precompetition period and after a real-life competition due to two reasons. Firstly, judokas simultaneously apply various techniques for body weight reduction. Most of the athletes reduce body weight according to their previous experiences. In a research conducted in laboratory settings, it was concluded that body mass reduction methods should be standardized. Secondly, in order to gain the desired weight, judokas do not have to significantly reduce their weight. Sometimes, a reduction below 2% of the initial body weight is sufficient [2]. Therefore, the study included athletes with different weight reduction values.

Most of the available reports [15–17] assessed the prooxidative-antioxidative status in athletes who reduce by more than 5% of their initial body weight. Kowatari et al. [18] proved that even lower weight reductions—below 5%—may modify redox balance. However, the study assessed only selected parameters of the prooxidative-antioxidative status. The influence of rapid weight loss before a real competition on the enzymatic, nonenzymatic, and antioxidative systems and lipid peroxidation markers in athletes differing in weight reduction values is still unestablished. Therefore, the main aim of the study was to assess the prooxidative-antioxidative processes during the precompetition period and after a real tournament wherein judokas rapidly reduce weight. We hypothesized that prooxidative-antioxidative processes may differ in regard to weight reduction value. The secondary aim was attempting to characterize the dependency of prooxidative-antioxidative processes on a weight reduction value.

## 2. Materials and Methods

### 2.1. Participants

Sample size was set with the usage of G Power 3.0.10 software. With the assumption of effect size *f* = 0.25, *α* = 0.05, and statistical power = 0.80 for three measurements in two groups, the sample size was established on 28 people. Because of a drop-out possibility, the study included 30 judokas.

The following inclusion criteria were set: age above 18 years, at least brown belt, regular trainings including 10 exercise units per week, and at least one expected competition with body weight reduction. Study protocol assumed the final measurement in a postcompetition period (7 days after the tournament). This is why inclusion criterion was also an adequate interval in body weight reduction. Judokas ingesting drugs or supplements potentially influencing oxidative stress parameters as well those with acute inflammation were excluded.

Potential participants were asked to provide a consent agreement conceding to the study's aim and protocol presentation. The study protocol was approved by the Bioethics Committee of the Medical University of Silesia in Katowice (resolution number: KNW/0022/KB1/82/14).

Eventually, the study included 30 judokas with an age range of 18-30 years (mean age: 22.4 ± 3.40 years). The participants trained in judo for 14.4 ± 3.8 years, whereas the medium number of trainings per week was 11.5 ± 0.5. Most participants (24 judokas) had a master level and black belts. 6 judokas had a trainee level (kyu grade) and brown belts. All participants reduce body weight regularly in a competition season. Weight loss was achieved by the application of self-selected methods including dietary restrictions (30 participants), fluid restrictions (29 participants), use of plastic clothing (22 participants), use of sauna (21 participants), and an increase in activities (15 participants).

### 2.2. Study Design

The study was longitudinal with three measurements. The first measurement was performed before body weight reduction began (two weeks before the competition). The second measurement was done before judokas were qualified to a weight category during the competition (one day before the competition). The final measurement was performed seven days after the competition, in a regeneration period.

### 2.3. Anthropometric Measurements

Body weight and composition assessment was estimated with the body composition analyzer Tanita BC-420MA based on bioelectric impedance analysis (BIA).

### 2.4. Blood Analyses

Blood sampling for biochemical analysis was performed between 8 and 9 a.m. after an overnight rest and at least a twelve-hour time interval from the last meal and exercise session. 10 ml of blood was taken from the antecubital vein. Blood was drawn into a tube filled with ethylenediaminetetraacetic acid to obtain blood plasma and cells to prepare hemolysates. The blood samples were centrifuged (15 min., 900 g, 4°C), immediately frozen, and stored at -20°C, until biochemical analyses could be performed.

#### 2.4.1. Assessment of Antioxidant Activity

The superoxide dismutase (SOD) activity and its two isoenzymes in plasma, copper- and zinc-dependent superoxide dismutase (Cu,Zn-SOD) and manganese-dependent superoxide dismutase (Mn-SOD), were measured by Oyanagui's method [19]. Enzymatic activity was expressed in nitrite units (NU) per milliliter of blood plasma.

The ceruloplasmin (CER) activity in plasma was determined spectrophotometrically by Richterich's method [20]. Enzymatic activity was expressed in milligrams per deciliter of blood plasma (mg/dl).

The catalase (CAT) activity was assayed in erythrocytes by Aebi's kinetic method [21] and expressed as units per milligram of protein (IU/mg protein).

Glutathione peroxidase (GPx) was measured in erythrocytes by Paglia and Valentine's kinetic method [22] and expressed as micromoles of NADPH utilized per minute and normalized to one gram of protein (IU/g protein).

Glutathione reductase (GR) was assayed by Richterich's kinetic method [20] and expressed as micromoles of NADPH utilized per minute and normalized to one gram of protein (IU/g protein).

Glutathione S-transferase (GST) was measured by Habig and Jakoby's kinetic method [23] and expressed as micromoles of thioether formed per minute and normalized to one gram of protein (IU/g protein).

Nonenzymatic antioxidant status was assayed with total antioxidant capacity (TAC) measured by Erel's method [24], and the concentration of protein sulfhydryl (PSH) was assayed by Koster et al.'s method [25]. TAC was expressed in mmol/g protein and -SH in *μ*mol/l.

#### 2.4.2. Determination of Prooxidative Status Parameters

Oxidative stress intensity was assayed with lipid hydroperoxides (LPH) which were measured by the method described by Södergren et al. [26], malondialdehyde (MDA) which was assayed by Ohkawa et al.'s method [27], and total oxidative stress (TOS) which was measured by the method introduced by Erel [28]. All concentrations were expressed as *μ*mol/l.

### 2.5. Statistical Analysis

Statistical analysis was performed with the SPSS ver. 25 software (IBM, Armonk, NY, USA). Descriptive statistics were presented for qualitative variables as numbers (*n*) and percentages (%), while quantitative variables were presented as mean (*M*) with standard deviation (SD).

One-way analysis of variance (ANOVA) with repeated measurements was performed to compare oxidative stress parameters between selected measurements. If statistical significance was found, the Least Significant Difference (LSD) post hoc test was applied. Relationships between weight reduction value and changes of oxidative stress parameters during the precompetition period were evaluated by regression analyses, including linear and curvilinear regression. The threshold for statistical significance was set at *p* < 0.05.

## 3. Results

In the precompetition period, judokas' weight reduction range was 0.44-6.10% (mean weight reduction: 2.93% ± 1.76%) of the initial body weight.  Table 1 presents changes of body mass index (BMI), body weight, and composition in pre- and postcompetition measurements. Between the first and the second measurement, statistically significant changes in BMI, fat mass (FM), muscle mass (MM), and total body water (TBW) were obtained in participants.

Changes of antioxidative parameters are presented in Table 2. Between the first and the third as well between the second and the third measurement, a statistically significant decrease in SOD, Mn-SOD, and CER occurred in participants. No significant changes of antioxidative status were observed in precompetition measurements.

Table 3 presents changes of prooxidative parameters. One day before the competition in all participants, a decrease of LPH concentration occurred. Between the initial and final assessment, a statistically significant decrease in concentrations of MDA, LPH, and TOS was observed.

**Figure 1:**
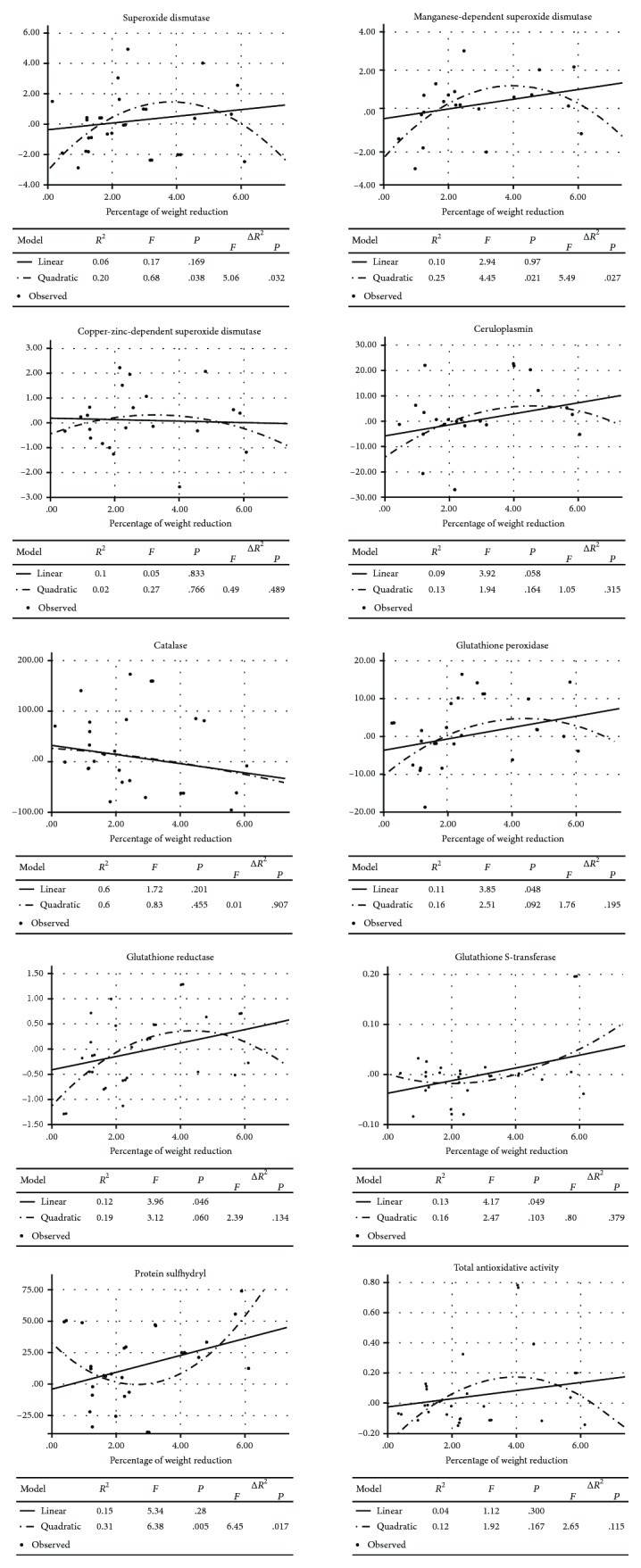
Weight reduction and antioxidant activity in judokas.

Regression models between the first and the second measurement are presented in Figure 1. Linear correlations were obtained for GPx, GR, GST, and PSH. A higher weight reduction was related to an increase in those parameters. Therefore, curvilinear regressions were obtained for SOD, Mn-SOD, and PSH. A moderate weight reduction induced the highest rise in SOD and Mn-SOD, while high and low reductions led to a lower one. An opposite correlation occurred in the case of PSH. Regression models for the remaining parameters of the antioxidative system were not statistically significant.

**Figure 2:**
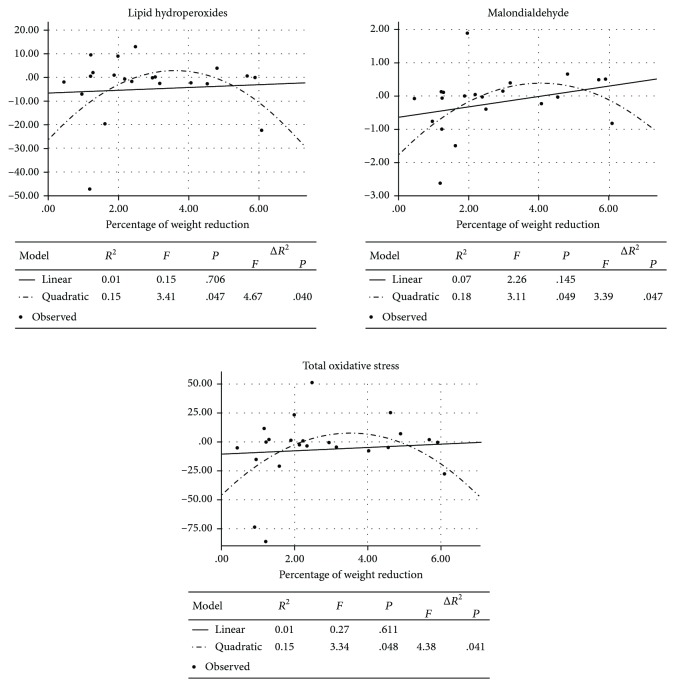
Weight reduction and prooxidative status in judokas.

Figure 2 contains regression models for prooxidative system changes between the first and the second measurement. Curvilinear regressions between a weight reduction value and oxidative stress parameters were obtained. A moderate weight reduction was correlated with a higher increase in MDA, LPH, and TOS, while low and high weight reductions led to a lower rise of those parameters.

## 4. Discussion

To the best of our knowledge, this is the first study that assessed the prooxidative-antioxidative status in pre- and postcompetition periods in judokas differing in weight reduction value.

In judokas, weight reduction in a precompetition period resulted in significant changes in the following body composition parameters: FM, MM, and TBW. A decrease in FM and MM might be a consequence of dietary restrictions and an increase in physical activity. Furthermore, simultaneously applied liquid restrictions and perspiration-stimulating methods might have resulted in a TBW decrease. It is difficult to compare our results with those available in the literature because of the diversity of protocols and assessment methods. Overall, the results of selected studies indicate that rapid weight loss decreases TBW [8, 29], FM [29], body fat percentage [8, 16], and free fat mass [16, 29]. Therefore, our results are in line with the cited studies.

Between the first and the second stage of the study, antioxidative system changes were observed but they were not statistically significant. Our study assessed different parameters of an antioxidative system from those available in the literature; thus, it is difficult to compare them. In accordance with our results, Rejic et al. [17] proved that rapid weight loss does not influence the serum levels of antioxidative vitamins A, C, and E and levels of GSH and GSSG in elite boxers. Therefore, Finaud et al. [16] observed that rapid weight loss increases the concentration of uric acid being an antioxidant. In our study, a lack of the antioxidative status changes in precompetition measurements may have several reasons. Participants opted for restrictions in food and liquid intake as well as the use of perspiration-stimulating methods to gain their desired weight. Similarly to Kowatari et al. [18] and Reljic et al. [17], we do believe that weight reduction might influence antioxidant capacity only on the condition that exercises mediate weight loss. On the contrary, it is well-established that an increase in antioxidative capacity is a physiological adaptation to regular physical activity [30]. Therefore, considering the fact that the study included high-level judokas, an exhausting physical effort would not have had influenced the concentration of antioxidants. Such hypothesis should be verified in further researches based on standardized weight reduction methods as well as in researches conducted in laboratory settings. Overall, the prooxidative-antioxidative system in athletes is more stable than that in nontraining people in response to strenuous conditions, e.g., exhausting physical exercises [30] and passive or active overheating [13]. However, it cannot be excluded that antioxidative status changes might be visible if judokas decided to increase the value of body weight reduction. Indeed, a linear regression model indicated that a higher weight reduction was related to the increased activity of GPx, GR, GST, and PSH. Perhaps, the weight reduction period was too short to induce antioxidative system changes.

Between the first and the second measurements, the concentration of LPH decreased significantly, while levels of MDA and TOS remained stable. On the other hand, linear regression models indicated that low and high weight reductions were related to a lower increase of MDA, LPH, and TOS rather than to a moderate weight reduction. Regression models of those parameters have a diverted letter “U” shape. Received results may be partially explained using a hormesis theory which claims that a potentially threatening factor applied in low doses may influence an organism beneficially. However, exceeding the body compensation capacity leads to harmful effects [31]. It is possible that a rapid loss of a low percentage of body weight (below 2%) limits the production of ROS and lipid peroxidation. What is interesting is that a reduction above 5% of the initial body weight has a similar effect. However, those results should be interpreted cautiously because in our study only three judokas decided to reduce weight so meaningfully.

Probably in judokas with a moderate weight reduction in response to elevated levels of LPH, MDA and TOS activity of antioxidative enzymes increased SOD and Mn-SOD. Curvilinear regression models of those parameters also have a diverted letter “U” shape. An opposite correlation was observed for a PSH concentration. A PSH curvilinear regression model has a diverted bell-shaped curve. It might indicate a decreased level of PSH as a consequence of an increased production of ROS in judokas with a moderate weight loss. In participants with weight reductions below 2% and above 5% of the initial body weight, PSH concentration increased while ROS production decreased. The obtained results may indicate that in judokas who reduce their weight PSH acts like a first-line-defense antioxidant. Indeed, PSH contained in blood plasma is an important nonenzymatic extracellular antioxidant being a scavenger of peroxides and an agent protecting neighbouring tissues from damage [25]. However, mutual interactions of pro- and antioxidative systems are extremely diversified, and our study confirmed that, too [32].

Our study proves that weight reduction influences prooxidative stress parameters divergently and is dependent on weight reduction value. However, it should be verified by further studies that include participants who have differing weight reduction values. Studies conducted by Finaud et al. [16] proved that 5% weight reduction does not induce lipid peroxidation. The discrepancy between our and Finaud et al.'s [16] results may be a consequence of the assessment of different lipid peroxidation markers. It cannot be excluded that a low-range weight reduction diminishes lipid peroxidation. We do believe that this process limitation may be associated with the application of dietary restrictions as the main weight reduction method. Indeed, it has been established that calorie restrictions in laboratory rodents inhibit lipid peroxidation. Generally, it is thought that dietary restrictions reduce the velocity of oxygen-dependent metabolic reactions with a secondary decrease in reactive oxygen species production [33, 34]. However, to our knowledge the influence of short-term calorie restrictions (one or two weeks) in athletes was not assessed. A recent study conducted by Pons et al. [35] revealed that six-week dietary restrictions significantly reduce the MDA concentration in athletes. However, as seen in our results, it is not obligatory to maintain dietary restrictions for six weeks to reduce the concentration of lipid peroxidation.

In the third stage of our study, antioxidative enzyme activities (SOD, Mn-SOD, and CER) as well LPH, MDA, and TOS concentrations were significantly lower than before competitions. Probably, their concentration reduction was a consequence of an intensive physical effort during the competitions. According to Nikolidis et al. [36], disturbances of the prooxidative-antioxidative status may persist even a couple days after strenuous exercises. However, a hypothesis that a decrease in SOD, Mn-SOD, and CER concentrations is a long-term consequence of rapid weight reduction cannot be rejected. Interestingly, one week after the competitions, the concentrations of LPH, MDA, and TOS were lower than in the first measurement. Probably, despite the reduction in levels of SOD, Mn-SOD, and CER, other antioxidants were effectively protected from a dynamic increase in ROS concentrations. However, we did not observe a significant rise in Cu,Zn-SOD, CAT, GPx, GR, GST, -PSH, and TAC levels.

Our study has some limitations. Firstly, only few judokas decided to undergo a high weight reduction. However, asking athletes before real-life competitions to reduce a higher percentage of their body mass despite having gained the desired weight would be risky and immoral. It might have a harmful influence on their competition success. Secondly, despite the fact that the study eliminated judokas ingesting drugs and supplements with potential effects on the prooxidative-antioxidative balance, we cannot exclude that the long-lasting effects of administering drugs and supplements before the study began might have determined the obtained results, particularly upon the first measurement. Thirdly, participants have maintained their routine training course during the study. Despite the fact the blood sampling was performed at least 12 hours after the last training, probably the first measurement did not display the resting condition of the blood redox homeostasis. Fourthly, despite that participants were requested to fill in the dietary diary, most of the judokas did not provide adequate feedback. We were unable to collect solid information about their calory and macro- and micronutrient intake. Finally, participants applied various weight reduction methods simultaneously. It is difficult to determine the influence of a particular weight reduction method on the prooxidative-antioxidative status. However, obligating the judokas to change their weight reduction strategy before real-time competitions might have harmful effects. That is why further studies should be conducted in laboratory settings with standardized weight reduction methods.

## 5. Conclusion

Summing up, our study reveals that the impact of weight reduction on prooxidative-antioxidative status is divergent and reduction-value dependent.

## Figures and Tables

**Table 1:** Participants' characteristics.

	Study stage			ANOVA
	I	II	III	
	Mean ± standard deviation			*F* (eta^2^)
Body weight (kg)	78.9 ± 16.1	76.6 ± 16.3	78.7 ± 15.7	**242.5** ^∗∗∗^ **(0.77)**
BMI (kg/m^2^)	24.78 ± 3.27	24.07 ± 3.37	24.75 ± 3.12	**161.75** ^∗∗∗^ **(0.85)**
FM (kg)	7.96 ± 5.49	7.65 ± 5.35	7.74 ± 5.46	**2.62** ^∗^ **(0.08)**
MM (kg)	65.69 ± 10.29	64.09 ± 10.67	65.74 ± 9.89	**47.16** ^∗∗∗^ **(0.63)**
TBW (kg)	48.21 ± 7.24	47.05 ± 7.64	48.32 ± 7.04	**33.21** ^∗∗∗^ **(0.54)**

Statistical significance: ^∗^*p* < .05; ^∗∗^*p* < .01; ^∗∗∗^*p* < .001. I—2 weeks before the competition; II—1 day before the competition; III—7 days after the competition. BMI—body mass index; FM—fat mass; MM—muscle mass; TBW—total body water. Statistical significance levels: body weight: *p* < 0.001; BMI: *p* < 0.001; FM: *p* = 0.042; MM: *p* < 0.001; TBW: *p* < 0.001.

**Table 2:** Antioxidative status parameters in judokas.

	Study stage			ANOVA
	I	II	III	
	Mean ± standard deviation			
SOD (NU/ml)	15.03 ± 5.07	15.32 ± 5.05	14.09 ± 5.90	**6.46** ^∗∗^ **(0.19)**
Mn-SOD (NU/ml)	9.56 ± 2.47	9.76 ± 2.01	8.41 ± 2.48	**15.29** ^∗∗∗^ **(0.35)**
Cu,Zn-SOD (NU/ml)	5.48 ± 3.32	5.56 ± 3.59	6.31 ± 5.64	ns
CER (mg/dl)	35.93 ± 11.16	36.43 ± 8.05	31.12 ± 8.11	**4.17** ^∗^ **(0.13)**
CAT (kIU/g Hb)	574.32 ± 119.63	580.11 ± 98.51	562.71 ± 108.93	ns
GPx (IU/g Hb)	54.58 ± 5.56	55.14 ± 6.39	54.81 ± 5.58	ns
GR (IU/g Hb)	5.41 ± 1.12	5.38 ± 1.04	5.32 ± 0.93	ns
GST (IU/g Hb)	0.16 ± 0.05	0.10 ± 0.06	0.16 ± 0.05	ns
-PSH (*μ*mol/l)	313.77 ± 62.84	325.92 ± 75.99	344.80 ± 154.59	ns
TAC (mmol/l)	1.08 ± 0.21	1.13 ± 0.21	1.12 ± 0.18	ns

Statistical significance: ^∗^*p* < .05; ^∗∗^*p* < .01; ^∗∗∗^*p* < .001; ns—nonsignificant results. I—2 weeks before the competition; II—1 day before the competition; III—7 days after the competition. Mn-SOD—manganese-dependent superoxide dismutase; Cu,Zn-SOD—copper- and zinc-dependent superoxide dismutase; CER—ceruloplasmin; CAT—catalase; GPx—glutathione peroxidase; GR—glutathione reductase; GST—glutathione S-transferase; PSH—protein sulfhydryl. Statistical significance levels: SOD: *p* = 0.004; Mn-SOD: *p* < 0.001; CER: *p* = 0.027.

**Table 3:** Prooxidative status parameters in judokas.

	Study stage			ANOVA
	I	II	III	
	Mean ± standard deviation			
LPH (*μ*mol/l)	12.71 ± 15.85	7.79 ± 5.96	4.82 ± 3.07	**5.55** ^∗∗^ **(0.17)**
MDA (*μ*mol/l)	2.62 ± 1.64	2.44 ± 1.40	2.12 ± 1.20	**4.25** ^∗^ **(0.13)**
TOS (*μ*mol/l)	23.10 ± 27.65	16.65 ± 16.31	10.31 ± 7.14	**4.16** ^∗^ **(0.13)**

Statistical significance: ^∗^*p* < .05; ^∗∗^*p* < .01; ^∗∗∗^*p* < .001; ns—nonsignificant results. I—2 weeks before the competition; II—1 day before the competition; III—7 days after the competition. LPH—lipid hydroperoxides; MDA—malondialdehyde; TOS—total oxidative stress. Statistical significance levels: LPH: *p* = 0.009; MDA: *p* = 0.012; TOS: *p* = 0.024.

## Data Availability

Result have not been published before. Supporting data may be provided by the corresponding author.
